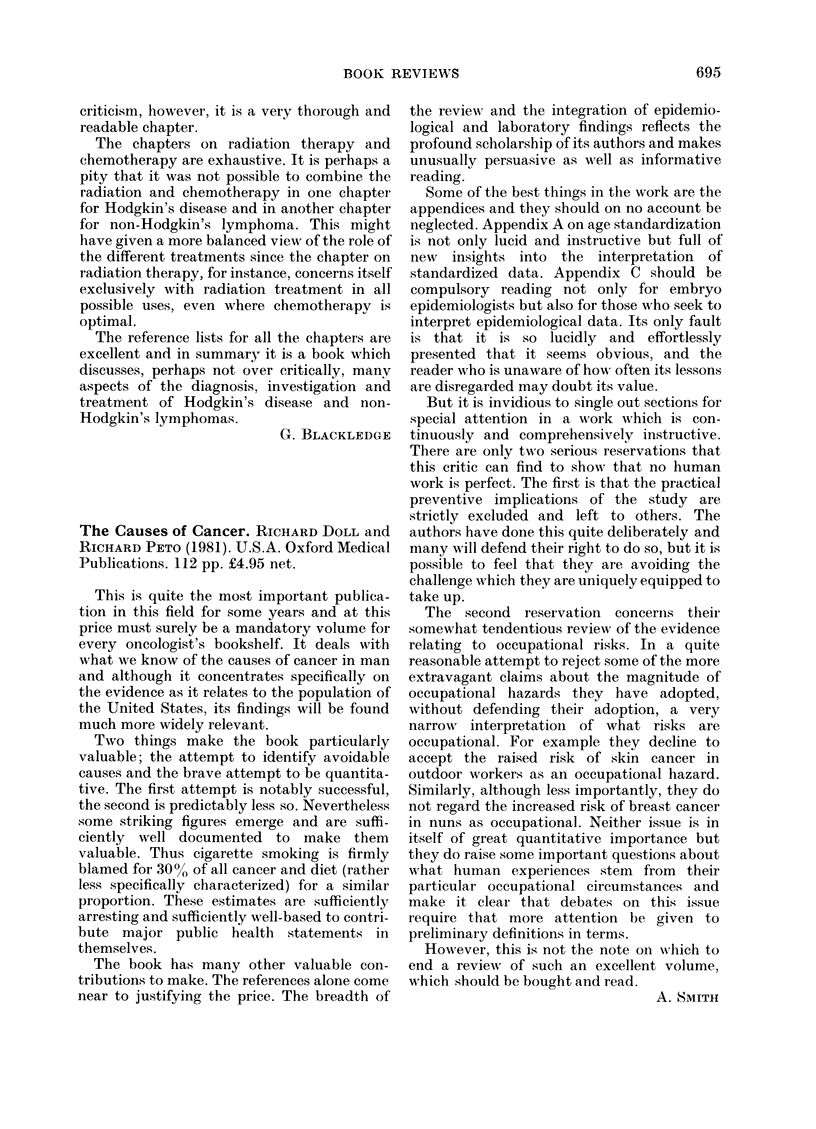# The Causes of Cancer

**Published:** 1982-10

**Authors:** A. Smith


**The Causes of Cancer.** RICHARD DOLL and RICHARD PETO (1981). U.S.A. Oxford Medical Publications. 112 pp. £4.95 net.

This is quite the most important publication in this field for some years and at this price must surely be a mandatory volume for every oncologist's bookshelf. It deals with what we know of the causes of cancer in man and although it concentrates specifically on the evidence as it relates to the population of the United States, its findings will be found much more widely relevant.

Two things make the book particularly valuable; the attempt to identify avoidable causes and the brave attempt to be quantitative. The first attempt is notably successful, the second is predictably less so. Nevertheless some striking figures emerge and are sufficiently well documented to make them valuable. Thus cigarette smoking is firmly blamed for 30% of all cancer and diet (rather less specifically characterized) for a similar proportion. These estimates are sufficiently arresting and sufficiently well-based to contribute major public health statements in themselves.

The book has many other valuable contributions to make. The references alone come near to justifying the price. The breadth of the review and the integration of epidemiological and laboratory findings reflects the profound scholarship of its authors and makes unusually persuasive as well as informative reading.

Some of the best things in the work are the appendices and they should on no account be neglected. Appendix A on age standardization is not only lucid and instructive but full of new insights into the interpretation of standardized data. Appendix C should be compulsory reading not only for embryo epidemiologists but also for those who seek to interpret epidemiological data. Its only fault is that it is so lucidly and effortlessly presented that it seems obvious, and the reader who is unaware of how often its lessons are disregarded may doubt its value.

But it is invidious to single out sections for special attention in a work which is continuously and comprehensively instructive. There are only two serious reservations that this critic can find to show that no human work is perfect. The first is that the practical preventive implications of the study are strictly excluded and left to others. The authors have done this quite deliberately and many will defend their right to do so, but it is possible to feel that they are avoiding the challenge which they are uniquely equipped to take up.

The second reservation concerns their somewhat tendentious review of the evidence relating to occupational risks. In a quite reasonable attempt to reject some of the more extravagant claims about the magnitude of occupational hazards they have adopted, without defending their adoption, a very narrow interpretation of what risks are occupational. For example they decline to accept the raised risk of skin cancer in outdoor workers as an occupational hazard. Similarly, although less importantly, they do not regard the increased risk of breast cancer in nuns as occupational. Neither issue is in itself of great quantitative importance but they do raise some important questions about what human experiences stem from their particular occupational circumstances and make it clear that debates on this issue require that more attention be given to preliminary definitions in terms.

However, this is not the note on which to end a review of such an excellent volume, which should be bought and read.

A. SMITH